# Advantages of using graph databases to explore chromatin conformation capture experiments

**DOI:** 10.1186/s12859-020-03937-0

**Published:** 2021-04-26

**Authors:** Daniele D’Agostino, Pietro Liò, Marco Aldinucci, Ivan Merelli

**Affiliations:** 1 Institute of Electronics, Computer and Telecommunication Engineering, National Research Council of Italy, Genoa, Italy; 2 Computer Laboratory, University of Cambridge, Cambridge, UK; 3 Computer Science Department, University of Turin, Turin, Italy; 4 Institute for Biomedical Technologies, National Research Council of Italy, Segrate, MI Italy

**Keywords:** Hi-C, Chromatin capture, Graph databases, Graph visualisation

## Abstract

**Background:**

High-throughput sequencing Chromosome Conformation Capture (Hi-C) allows the study of DNA interactions and 3D chromosome folding at the genome-wide scale. Usually, these data are represented as matrices describing the binary contacts among the different chromosome regions. On the other hand, a graph-based representation can be advantageous to describe the complex topology achieved by the DNA in the nucleus of eukaryotic cells.

**Methods:**

Here we discuss the use of a graph database for storing and analysing data achieved by performing Hi-C experiments. The main issue is the size of the produced data and, working with a graph-based representation, the consequent necessity of adequately managing a large number of edges (contacts) connecting nodes (genes), which represents the sources of information. For this, currently available graph visualisation tools and libraries fall short with Hi-C data. The use of graph databases, instead, supports both the analysis and the visualisation of the spatial pattern present in Hi-C data, in particular for comparing different experiments or for re-mapping omics data in a space-aware context efficiently. In particular, the possibility of describing graphs through statistical indicators and, even more, the capability of correlating them through statistical distributions allows highlighting similarities and differences among different Hi-C experiments, in different cell conditions or different cell types.

**Results:**

These concepts have been implemented in NeoHiC, an open-source and user-friendly web application for the progressive visualisation and analysis of Hi-C networks based on the use of the Neo4j graph database (version 3.5).

**Conclusion:**

With the accumulation of more experiments, the tool will provide invaluable support to compare neighbours of genes across experiments and conditions, helping in highlighting changes in functional domains and identifying new co-organised genomic compartments.

**Supplementary Information:**

The online version contains supplementary material available at 10.1186/s12859-020-03937-0.

## Background

Modern bioinformatics aims at integrating different omics data to shed light into the mechanisms of gene expression and regulation that give rise to different phenotypes, in order to understand the underlying molecular processes that sustain life and to intervene into these processes by developing new drugs [1, 2] when pathological changes occur [3, 4]. In this context, the exploration of the 3D organisation of chromosomes in the nucleus of cells is of paramount importance for many cellular processes related to gene expression regulation, including DNA accessibility, epigenetic patterns, and chromosome translocations [5, 6]. The 3D chromatin analysis will likely provide an effective and standard diagnostic methodology for cancer metastatic clones and genetic diseases. Although the following description focuses on the analysis and visualisation of High-throughput sequencing Chromosome Conformation Capture technique, the approach is general and can work with other chromatin capture technologies.

High-throughput sequencing Chromosome Conformation Capture (Hi-C) technique allows the study of chromatin interactions and 3D chromosome folding on a larger scale [7, 8]. The graph-based representation of Hi-C data produced, for example, by NuChart [9, 10] or CytoHic [11], which are software for representing the spatial position of genes in the nucleus, are essential for creating maps where further omics data can be mapped, in order to characterise different spatially associated domains. This represents an effective complement of the traditional matrix-based representation, as for example produced by Juicer [12] or TADbit [13].

Contact matrices, or better their probabilistic models, allow creating representations that only involve two chromosomes, while graphs can describe the interactions of all the chromosomes using a graph-based approach. This representation highlights the physical proximity of genes in the nucleus in comparison to coordinate-based representations. The very same problem impairs representations based on Circos, which can characterise the whole genome in one shot, but fail to describe the physical proximity of genes.

Previous works [14, 15] show interesting results on the possibility of creating metrics for defining how far two genes are one from the other, with possible applications to cytogenetic profiling, to the analysis of the DNA conformation in the proximity of the nucleolus, and for describing the social behaviour of genes.

However, the typical size of a graph achieved through a Hi-C analysis is in the order of thousands of nodes and hundreds of thousands of edges, which makes its effective management and exploration too complicated. The critical aspect is the fact that the information about the relationships among the entities represented by the vertices is nearly as crucial as the vertices themselves. The relevance of this aspect is increasing in many applicative fields, as discussed in [16], and in particular in molecular biology [17] and bioinformatics [18].

Despite widely used repositories as STRING [19] or InterMine [20] are still based on SQL databases, many other platforms integrating heterogeneous bioinformatics repositories exploits graph databases, as BioGraphDB [21], Bio4j [22], biochem4j [23], or domain-specific repositories as Reactome for biomolecular pathways [24]. The list of the most important ones is provided in [17].

The performance in retrieving data represents a key feature. For example, graph databases like Neo4J outperform SQL-based systems like PostgreSQL in path discovery operations by several orders of magnitude [18].

On the other hand, visualisation remains so far an important issue. Most of the above platforms rely on general-purpose tools like esyN [25] or the well-known Cytoscape [26] for displaying the networks resulting from a query. However, these solutions are not effective in visualising the complexly structured Hi-C data. For this reason, some ad hoc analysis and visualisation tools have been developed as the R-based HiCeekR [27] or the NeoHiC web app [28].

NeoHiC supports the progressive visualisation and analysis of Hi-C data produced by NuChart, but it is straightforward to ingest data produced by other tools. It relies on the use of the Neo4j graph database, its graph data science framework, and modern web technologies as Node.js, widely used in many scientific applications [29]. This paper is an extended version of [28], where only the progressive visualisation aspects have been presented. Here the focus is in the use of the Neo4j Graph Data Science framework for analysing and comparing the relationships and network structures of available experiments.

## Methods

NeoHiC retrieves the data stored in a Neo4j database instance for their analysis and visualisation. In particular, while the visualisation represents a gene-centric exploration of the experiments, it is also possible to compute statistic values about the significance of some graph characteristics, which is an experiment-centric analysis representing a description of all the components of the system.

### The data

The output of a Hi-C analysis is a list of paired genomic regions along the different chromosomes, which can be represented as a square matrix X, where $X_{ij}$ stands for the sum of read pairs matching in position *i* and position *j*, respectively. This representation is called a *contact map* and it focuses on providing a measure of the contact frequencies between groups of genomic bins. The frequency values rely on bins spatial proximity and, therefore, are related to their distance. While a contact map is reliable for looking at the intensity of the interactions inside a chromosome or between two chromosomes, it becomes unsuitable for depicting the neighbourhood of a gene (or of a cluster of genes), which may involve multiple chromosomes. This gene-centric view is of particular interest for making Hi-C experiments a common ground for integrating multi-omics features, highlighting, in systems biology view, pathways and transcriptional programs regulated by the genome conformation.

On the contrary, graph-based representation of Hi-C data has a high level of expressiveness, because its structural properties can reveal important information on how the actors of the represented process, i.e. the genes, interact. This is the reason why NeoHiC has been designed for the visualisation and analysis of these graphs produced by tools able to provide a gene-centric representation, like NuChart. This software creates graphs in which the vertices are the genes, and the edges are the Hi-C contacts represented by the reads as provided by the Hicup software [30]. In particular, an edge identifies the presence of a Hi-C valid read that encompasses two connected genes. The number of reads supporting contact evidence, which is an of the interaction strength, is then used as the weight of the edge, working as a proxy of the physical closeness for the considered genes. Edges can be further characterised by assigning them scores related to genomic and epigenomic features, which may include regulatory patterns, methylation profiles, histone modifications and other genomic structural landmarks.

### The database

Graph databases are part of the NoSQL database family created to address some issues of the entity-relational data model. While the graph model explicitly lays out the dependencies among the nodes, which represents the entities, the relational and other NoSQL database models link these entities through implicit connections.

In particular, in relational databases, references to other rows and tables are indicated by referring to primary key attributes via foreign key columns. Joins are computed at query time by matching primary and foreign keys of all rows in the connected tables. These operations are compute- and memory-intensive, having an exponential cost. Moreover, when many-to-many relationships occur in the model, there is the need to introduce a JOIN table (or associative entity table) that holds foreign keys of both the participating tables, further increasing storage space, and the execution time of join operations.

On the contrary, in the data model of graph databases, the relationships have the same importance as the nodes. Database designers are not required to infer connections among entities using special properties such as foreign keys. For this, graph databases, by design, allow fast and straightforward retrieval of complex hierarchical structures that are difficult to model in relational systems.

The most important concepts in a graph model like Neo4j are:

**Nodes**: represent the entities of the database.
**Labels**: are used to group nodes. There may be several labels associated with a node.
**Relationships**: connect pairs of a node. They are directed, even if it is possible to disregard this information.
**Types**: are associated with relationships, but a relationship has one and only one type.
**Properties**: are name-value pairs that can be associated with nodes and relationships.

Therefore, the first step for supplying a new Hi-C experiment is represented by its insertion in Neo4j. This is achieved by using a Javascript program, available with NeoHiC on GitHub, for converting the graph-based representation of Hi-C data in a set of CSV files that can be directly imported in Neo4j. In detail, this program parses the file produced by NuChart containing the list of edges and produces two files, one containing information about the experiment, the second the list of edges with their attributes.

Genes with their static information (i.e. the chromosome they belong to and the position) are stored once in the database as nodes. Also each experiment is represented as a node, with some statistical information as the number of genes and edges it contains. The edge file, instead, creates new relationships among genes, labelled with the name of the experiment. This means that a node representing a gene is created only once, then used as in all the edges involving it.

All the queries in Neo4j follow the Cypher graph query language. For example each edge is created using a query like the following one:

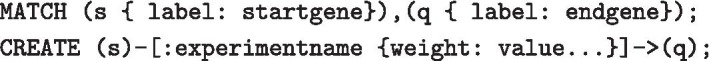



**Fig. 1:**
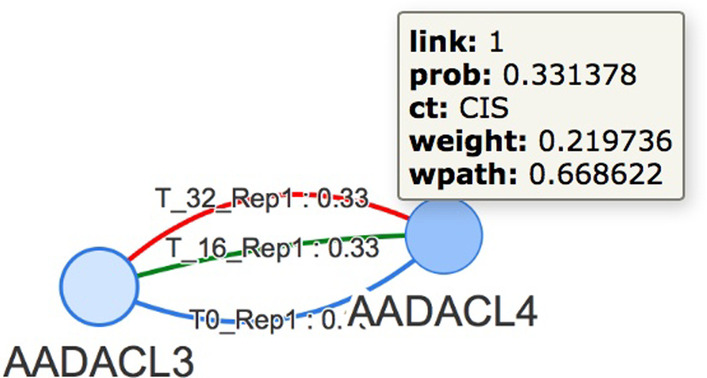
Graph example. An example of Hi-C data stored in Neo4j, which is composed by two nodes, the genes, AADACL3 and AADACL4, linked by three edges corresponding to experiments ’T0_Rep1’, ’T_16_Rep1’ and ’T_32_Rep1’. Each edge has 5 properties

The first line is used to retrieve the references of the two extreme genes; then they are used to create a link labelled with a name related to the experiment. An example of edges belonging to different experiments linking a pair of genes is shown in Fig. 1.

**Fig. 2:**
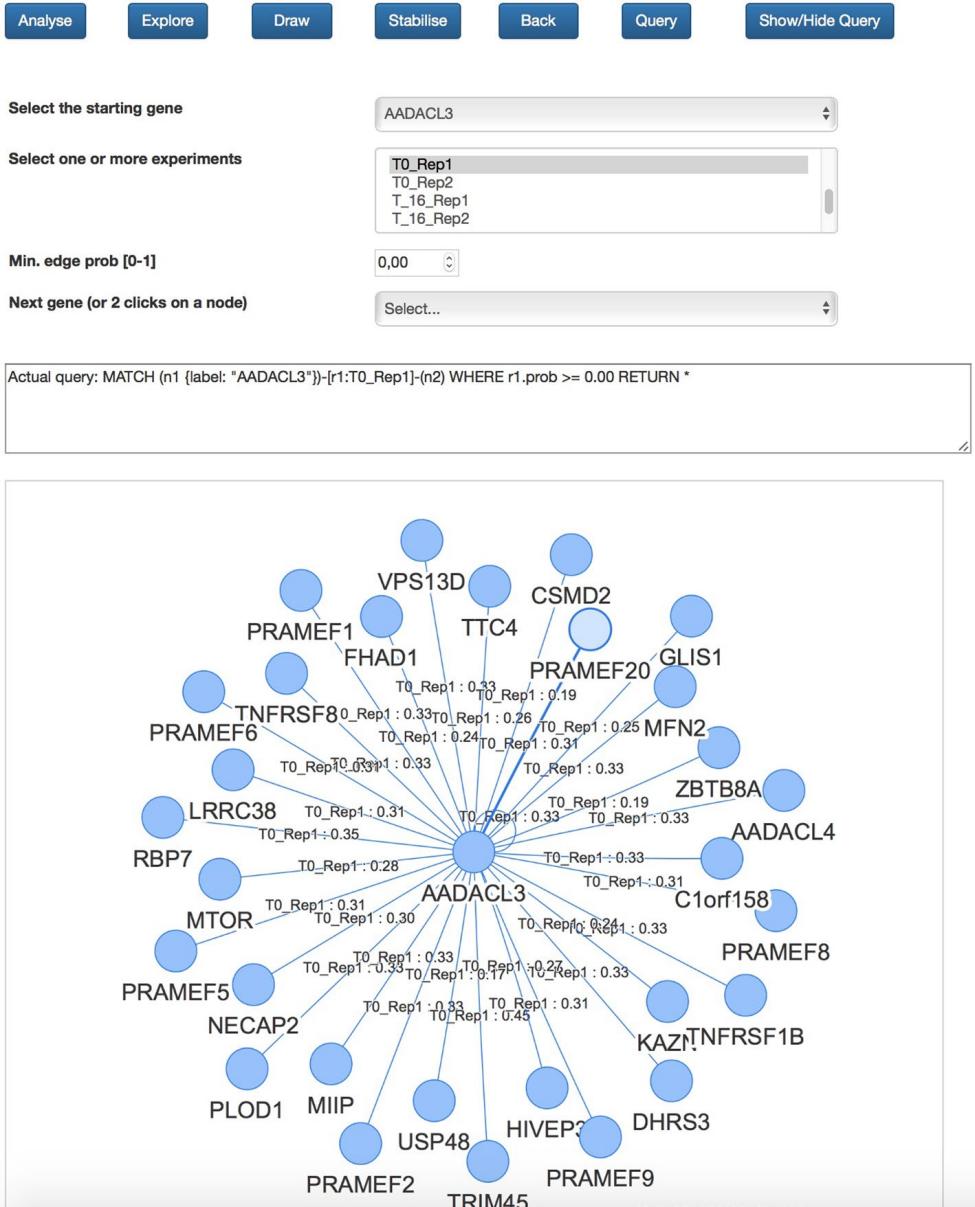
Hi-C data exploration. The NeoHiC graphical user interface for visualizing and exploring Hi-C networks starting from a gene

**Fig. 3:**
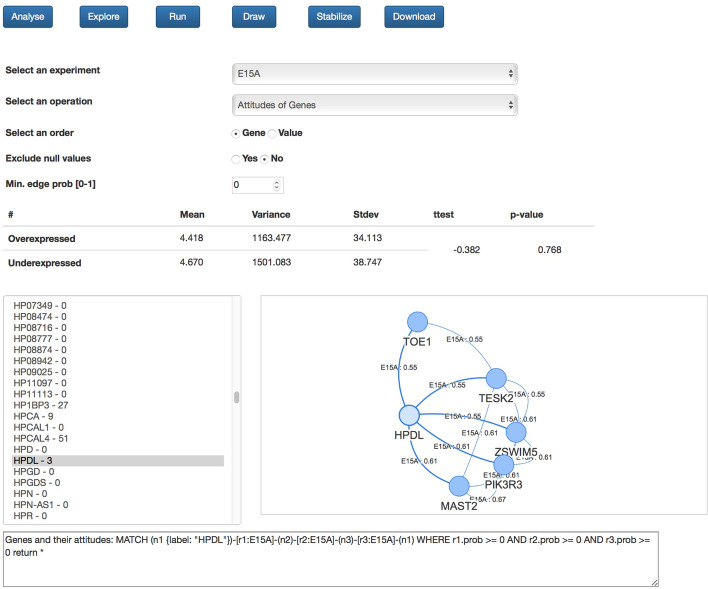
Hi-C data analysis. The NeoHiC graphical user interface for the statistical analysis of Hi-C networks starting from an experiment

**Fig. 4:**
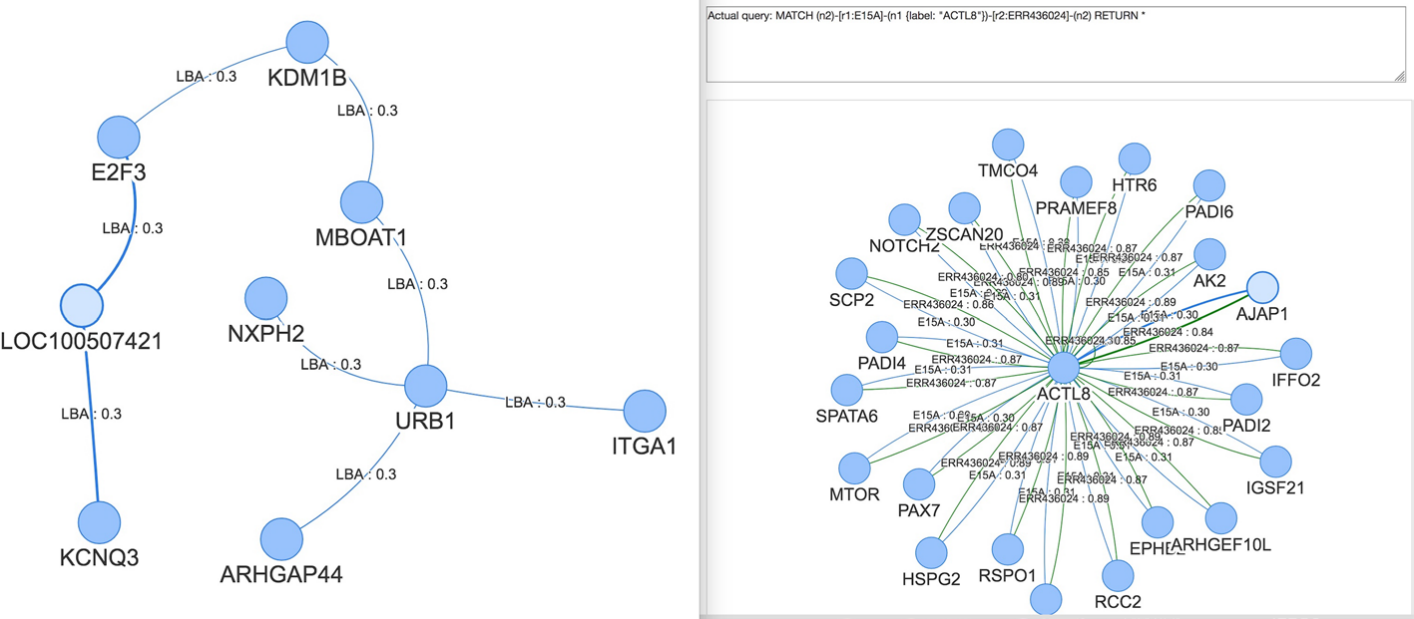
Visualisation example. The left side of the image show an example of progressive visualisation of a Hi-C graph. The right side the result of a user-defined query

**Fig. 5:**
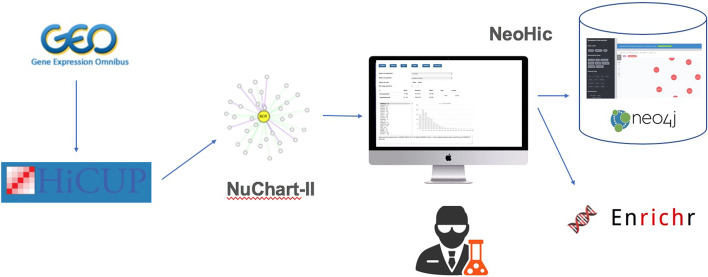
Data Analysis Workflow This is the Flowchart of the Hi-C data analysis using NeoHiC

**Fig. 6:**
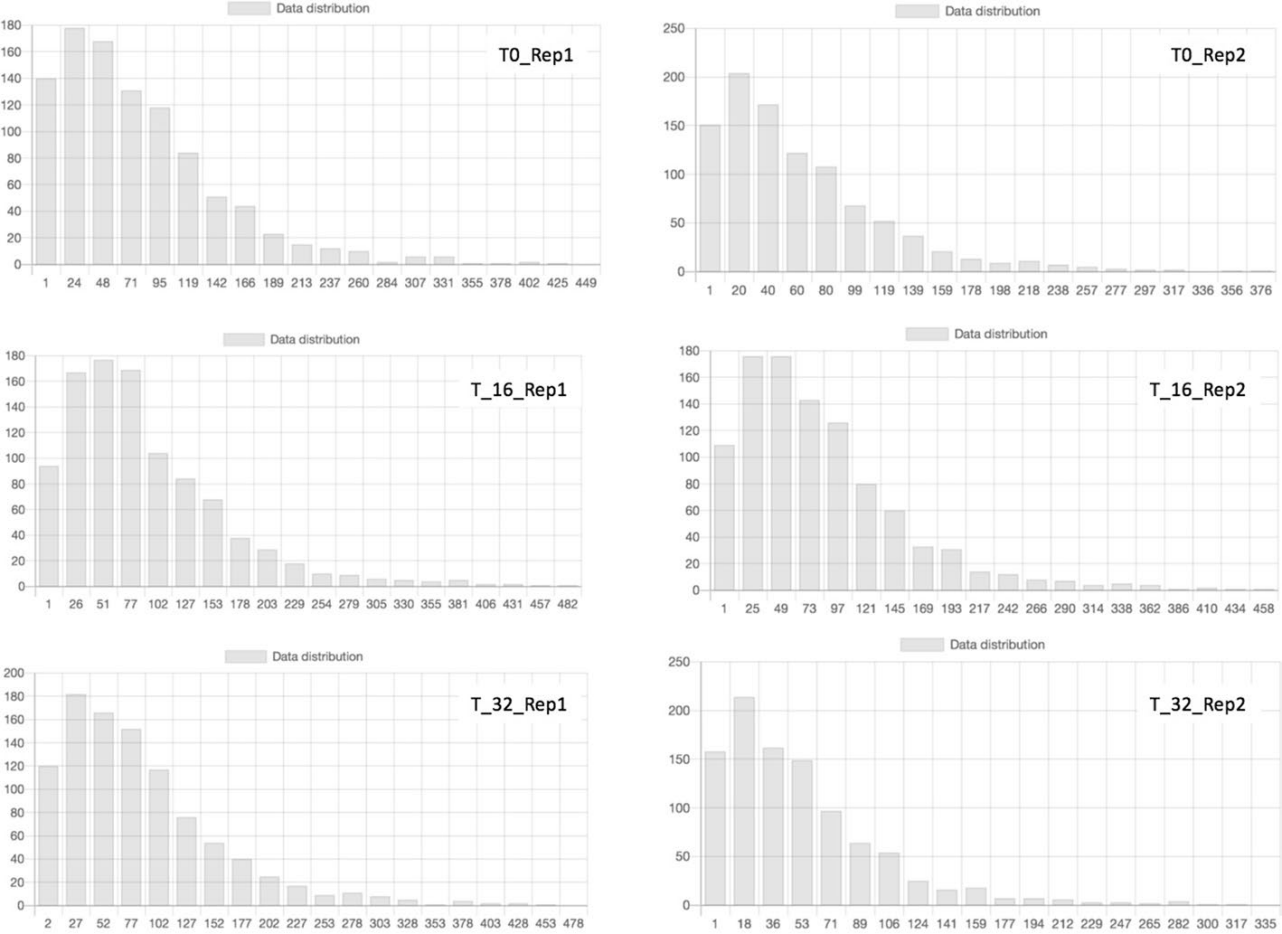
Gene degree values Distribution of genes degrees among the time series of the Hi-C experiments

**Fig. 7:**
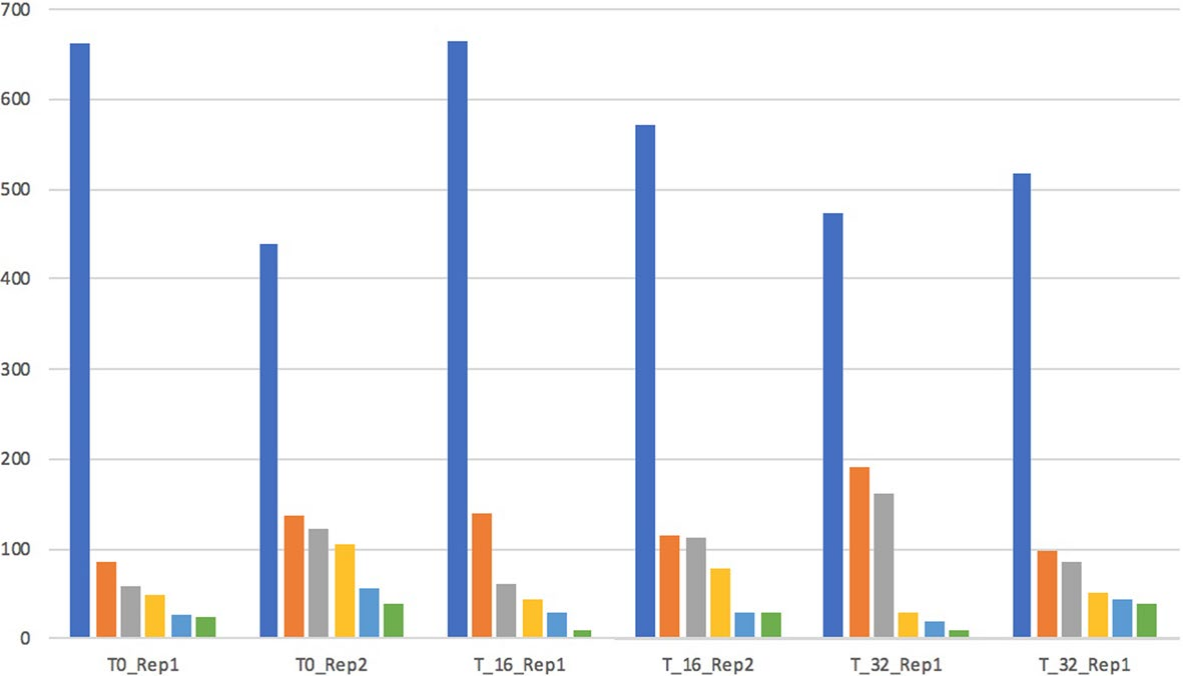
Louvain algorithm The clusterisation performed by the Louvain algorithm among the time series of the Hi-C experiments

**Fig. 8:**
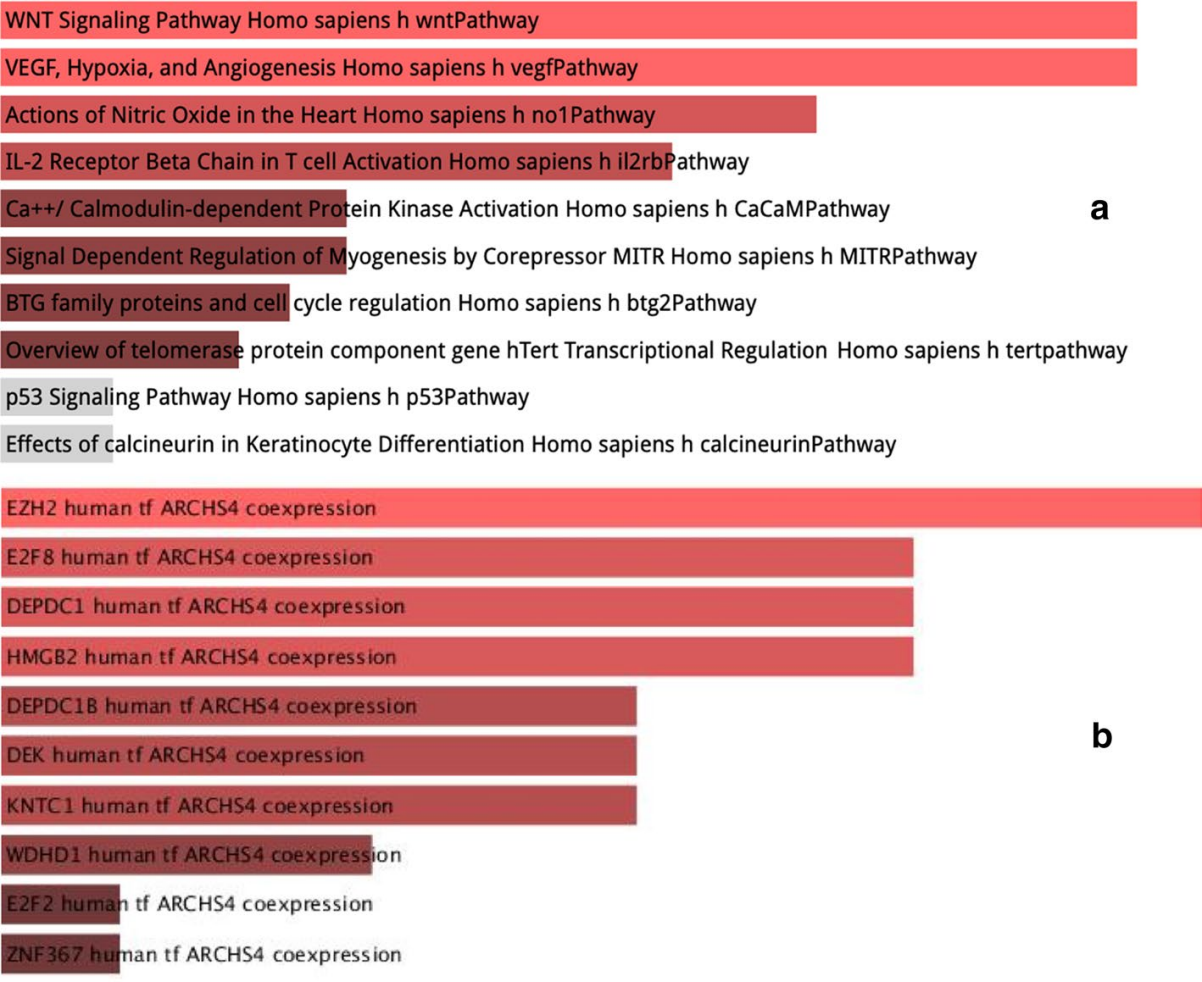
Enrichment analysis Panel A shows the enrichment analysis of genes presenting a different clustering attitude between the first and last time point. Panel B shows the enrichment analysis of the largest cluster identified by using the Louvain algotihm in the last time point of the Hi-C experiments

**Fig. 9:**
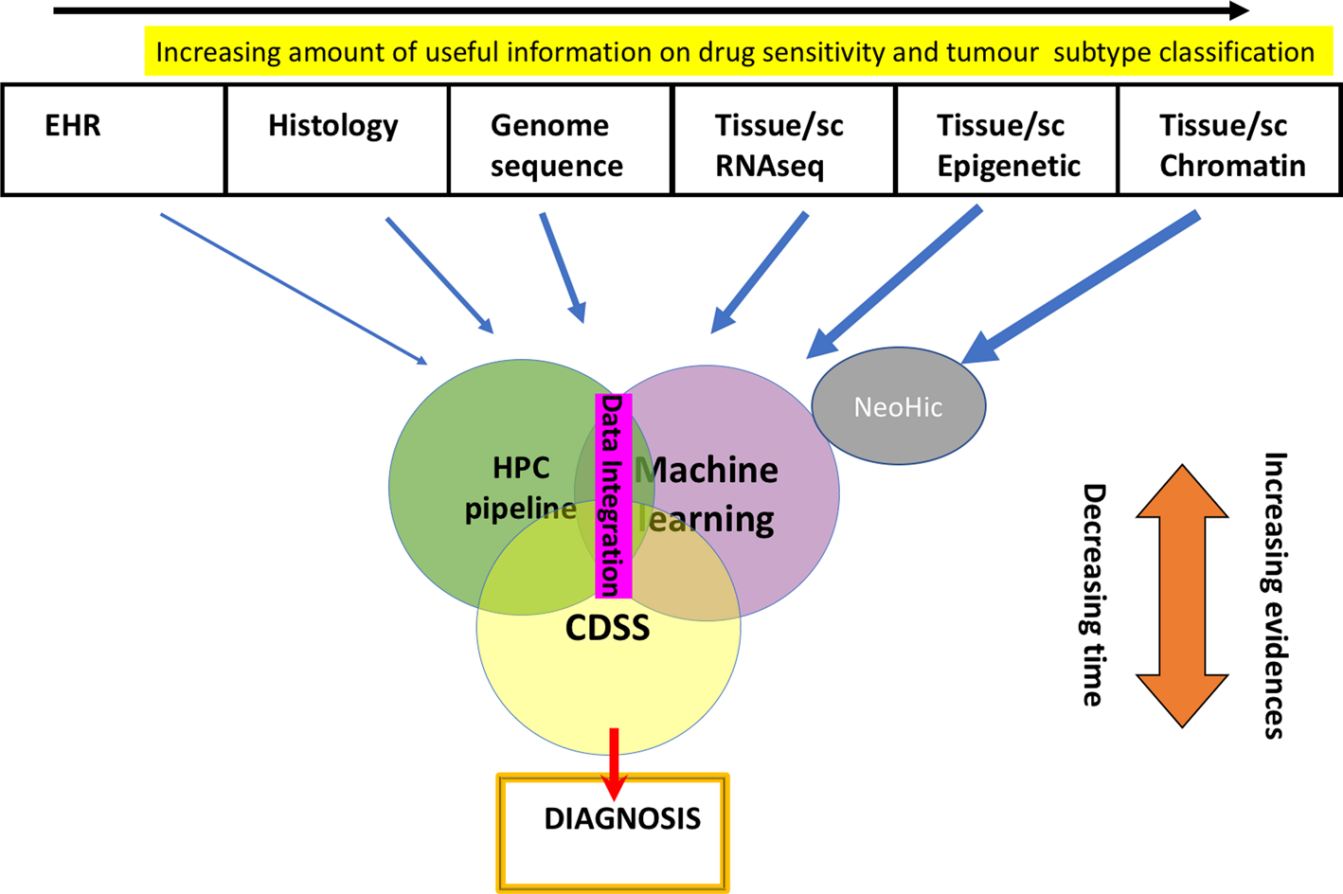
NeoHiC in the big picture NeoHiC can be a useful tool in the context of multi-modal data integration approaches, involving clinical data, histological information and multi-omics profiling, in order to develop improved Clinical Decision Support Systems

The Neo4j database can be installed on the user workstation or it is possible to exploit an instance available via Web.

### Hi-C visualisation

NeoHiC is a Web application written in Javascript and based on a customised version of the Neovis.js graph visualisation library (version 1.14) [31], which in turn mixes the Neo4j Javascript driver and the general-purpose vis.js library.

The application can be launched on the user workstation, or it is possible to exploit it via the Web. The Web app provides both the exploration mode and the analysis mode, as shown respectively in Figs. 2 and 3.

The exploration mode supports a gene-centric analysis of the experiments, because it starts with the selection of a gene and one/all/a set of experiments to be considered in showing neighbouring genes.

NeoHiC is based on the same approach adopted by STRING, where a protein-protein interaction network is expanded one step at a time by clicking on one of the visible nodes. Considering that a gene can have hundreds of neighbours, the user can expand the network also selecting genes from a list: all the genes connected to the selected ones will appear in the graph. These steps for expanding the network can be iterated many times as desired and the only information hold by the web app is represented by the query string, in Cypher, that is used to retrieve the data at every expansion by interacting with the Neo4j database. In particular, the example is shown on the left side of Fig. 4 corresponds to the following query
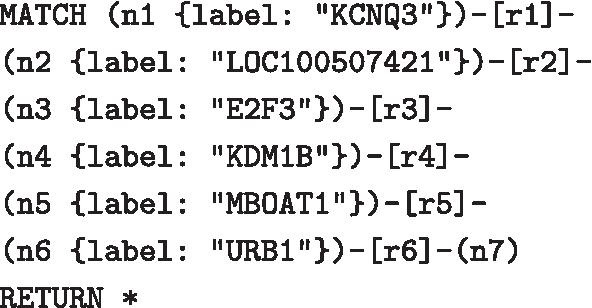


 where each of the five gene selections corresponds to add a $n_x-[r_x]-n_{x+1}$ pattern.

Expert users are allowed to modify the query or insert a new query to interact directly with Neo4j. An example is shown on the right side of Fig. 4, which shows the result of a manually-inserted query that filters, among the 262 neighbouring genes of “ACTL8”, only those with one link for each of the two selected experiments.

It is also possible to go back of one step with the *Back* button or of multiple steps by clicking on one edge, for example, on edge between “KDM1B” and “MBOAT1”, to provide the user with the possibility to freely navigate the graph. At last, it is possible to specify a threshold to filter the neighbouring genes based on the weight associated with them, corresponding to their probability as computed by NuChart.

### Hi-C analysis

The possibility of describing graphs through statistics and, even more, the capability of correlating them, represents an effective way to highlight similarities and differences in different Hi-C runs, in different cell conditions or in different cell types. For example, it is interesting the possibility to compute simple statistics about the significance of some graph characteristics, such as the topology of the edges, the vertex tendency to be reciprocal, the distribution of the vertex degree in the graph, the measure of the clustering attitude of each vertex, and centrality measures (such as betweenness and closeness) that describe in detail the neighbourhood of each gene of the graphs.

NeoHiC supports such experiment-centric analysis with the interface shown in Fig. 3. The implementation of the provided operations is based on the use of the Neo4j graph data science library. The library provides five classes of algorithms:

**Community Detection**: to detect groups of nodes having more significant interactions. NeoHiC supports the clustering of genes within an experiment using the Louvain algorithm.
**Similarity**: to score how alike nodes are based on their neighbours or other properties. NeoHiC includes the possibility to compute the Jaccard coefficient of a gene considering two subgraphs deriving from two experiments.
**Centrality**: to evaluate the importance of some genes based on graph topologies. NeoHiC supports Page Rank that measures the influence of a gene in paths randomly traversing the graph counting the frequency of hitting it; Betweennesses to detect genes that act as a bridge between parts of a graph; Closeness to detect genes having the shortest distances to all other genes.
**Path finding**: to compute the shortest path between two or more nodes. NeoHiC identifies and shows the shortest path between a pair of genes in an experiment or presents the shortest path from a gene to all other ones in an experiment. This last can be computed considering the number of links connecting the genes or also the probability associated to the edges by NuChart.
**Link prediction**: to evaluate the closeness of a pair of nodes in order to predict new relationships between them. We are working on extending the NeoHiC functionalities to exploit these algorithms to infer hidden relationships among genes that have not been captured by experiments.

Beside this library it is possible to exploit other external libraries, as the APOC—Awesome Procedures on Cypher one, including over 450 standard procedures, and the possibility to enrich genes using services as Enrichr [32, 33].

It is worth to note that NeoHiC is an extensible tool because it is straightforward to retrieve the raw information provided by a query in Cypher for their analysis using custom functions. Moreover, NeoHiC allows downloading gene lists and corresponding values as CSV files.

At last, NeoHiC can integrate other multi-omic datasets, as time series RNA-seq data or methylation profiles, by providing correlations between the graph structure and up-regulated/methylated or down-regulated/methylated genes. An example is shown in Fig. 3.

## Results

In this section, we discuss some results achieved using NeoHiC on a time series Hi-C analysis of breast cancer, since higher-order chromatin structures are often perturbed in cancer and other pathological states.

We tested NeoHiC on a publicly Hi-C dataset related to breast cancer [34], available at the Gene Expression Omnibus repository, with accession number GSE130916. This dataset is a time series of Hi-C experiments, intended to study changes in the DNA conformation of breast cancer cells of Estrogen Receptor (ER) positive patients that develop resistance and relapse after treatment, in order to understand the mechanisms underpinning endocrine resistance in this tumour. In particular, Hi-C was conducted in endocrine-sensitive breast cancer cells (MCF7) at three time-points during long-term culture: time zero (T0), mid-time point (T16) and late time point (T32, > 6 months). Two replicates are available for each time point.

The original study shows that chromatin interactions, both within and between topologically associating domains (TADs), frequently change in resistant breast cancer cells and that alterations in active (A-type) and inactive (B-type) chromosomal compartments are associated with decreased ER binding and atypical interactions and gene expression.

We downloaded the data from GEO, and we aligned the reads to the reference genome using HiCup, providing to the software the in-silico digested genome using the NcolII restriction enzyme. Then we computed the Hi-C graphs using NuChart as we loaded all the data inside the graph database. Then, using NeoHiC, we were able to deepen the analysis using a gene-centric view. The workflow is shown in Fig. 5. and it is described in details is the Additional file 1.

First of all, we computed the Jaccard distance between all the samples available in the datasets, as reported in Table 1. As we can see, considering the overall distribution of the genome-wide contacts, the reproducibility of the experiments is relatively low, and variations among the datasets are comparable with the variation between the different time points. Then, NeoHiC was used to compute the degree distribution for each dataset, plotting a barplot as reported Fig. 6. We tested this feature on our breast cancer test case and, as we can see in the figure, also, in this case, the overall distribution is quite similar in all the experiments, highlighting that the changes among the different conditions cannot be seen at the genome-wide level, but it should be analysed locally.

**Table 1:** Comparison of Jaccard values of the experiments

	T0_Rep1	T0_Rep2	T_16_Rep1	T_16_Rep2	T_32_Rep1	T_32_Rep1
T0_Rep1	1.0	0.32039	0.25954	0.36207	0.29688	0.30208
T0_Rep2	0.32039	1.0	0.24167	0.33962	0.32743	0.31325
T_16_Rep1	0.25954	0.24167	1.0	0.26667	0.26950	0.23214
T_16_Rep2	0.36207	0.33962	0.26667	1.0	0.29323	0.31000
T_32_Rep1	0.29688	0.32743	0.26950	0.29323	1.0	0.28704
T_32_Rep1	0.30208	0.31325	0.23214	0.31000	0.28704	1.0

**Table 2:** Comparison of Attitude values of the experiments using an RNA expression file

	Mean		Variance		Stdev		ttest	p-value
	Over	Under	Over	Under	Over	Under		
T0_Rep1	57.414	65.183	148,768.556	260,483.369	385.741	510.414	− 0.957	0.514
T_Rep2	35.804	40.533	64,094.867	110,165.476	253.193	331.937	− 0.892	0.536
T_16_Rep1	78.072	87.453	272,795.286	435,472.788	522.346	659.953	− 0.876	0.542
T_16_Rep2	66.021	73.790	197,558.089	335,109.194	444.516	578.929	− 0.838	0.556
T_32_Rep1	71.788	78.214	233,673.355	368,972.989	483.442	607.477	− 0.650	0.633
T_32_Rep1	24.017	27.524	28,116.175	49,032.629	167.694	221.450	− 0.994	0.502

**Table 3:** Comparison of Closeness values of the experiments using a methylation file

	Mean		Variance		Stdev		ttest	p-value
	Over	Under	Over	Under	Over	Under		
T0_Rep1	0.022	0.024	0.011	0.011	0.103	0.107	− 0.663	0.627
T0_Rep2	0.021	0.023	0.010	0.011	0.099	0.103	− 0.689	0.616
T_16_Rep1	0.023	0.024	0.011	0.012	0.105	0.110	− 0.324	0.800
T_16_Rep2	0.022	0.024	0.011	0.012	0.104	0.108	− 0.657	0.630
T_32_Rep1	0.022	0.024	0.011	0.012	0.104	0.108	− 0.657	0.630
T_32_Rep1	0.021	0.022	0.009	0.010	0.097	0.100	− 0.353	0.784

With this aim, by employing an ERGMs approach already available in the NuChart R package, it is possible to statistically analyse the structure of the local neighbourhood graph, implementing a stochastic model of the network and using MCMC to create an estimator trough a likelihood function. These models can be used to compute simple statistics about the significance of some graph characteristics, such as the topology of the edges, the vertex tendency to be reciprocal, the distribution of the vertex degree in the graph, or the measure of the clustering attitude of each vertex.

Using this approach, we discovered that gene-rich chromosomes, such as chr16 through chr22, in the latest time point display decreased interaction frequency with each other compared to the inter-chromosomal interaction frequency in the earliest time point. Using enrichment analysis with the Biocarta 2016 database, we identified that involved genes are related to pathways of WNT signalling (see Fig. 8a).

There are also differences in intra-chromosomal interactions in telomeric regions since cells display more progressively fewer interactions time point after time point (see again Fig. 8a). These pieces of evidence are in agreement with previously reported, although in with a different experimental design [35].

Relying on the hypothesis that changes are locally more important, we also used the Louvain algorithm to identify clusters of genes in the different experiments. As reported in Fig. 7, there are significant changes in the distribution of the clusters. Using enrichment analysis (using the WikiPathway 2019 database), we identified some clusters of genes enriched for pathways related to mammary carcinoma and EZH2 pathways, as reported in Fig. 8b.

NeoHiC can also be used to integrate many different multi-omic datasets. In particular, we considered time-series RNA-seq data from estrogen-responsive breast cancer [36] in order to verify if genes that are up-regulated or down-regulated clusters deferentially. Using NeoHiC, we computed Table 2, reporting Mean and Variance of the clustering attitude of the up-regulated and down-regulated genes and the related p-value computed using a single t-test. As we can see, the clustering attitude has little difference in the different experiments.

We also used NeoHiC to integrate the Hi-C experiments with methylation [37], which is an important mechanism of tumour relapse. We computed the probability of having differences in the closeness of genes in the graph due to different methylation profiles. Results are reported in Table 3. Also, in this case, we can see that genome-wide there are no deep changes in the conformation of the chromatin dissected relying on methylation patterns.

The absence of a significant impact from methylation combined with the identification of increasing importance of the EZH2 pathway may suggest that the relapse is following a polycomb related mechanism of immune escape [38].

## Discussion and conclusion

NeoHiC is an extensible Web app that supports the efficient analysis and exploration of Hi-C data. NeoHiC requires only to access to a Neo4j database, which might run on the same machine of the application or as Software-as-a-Service (SaaS) in the cloud. The performance of NeoHiC depends on three factors. (1) the capabilities of the server hosting the Neo4j database because it is responsible for the data extraction and aggregation operation; (2) the bandwidth for the data transfer, whose size is however in the order of a few megabytes also for visualising large neighbourhoods made up by 1,000 genes; (3) the user device for the data visualisation because NeoHiC is a Javascript-based application.

NeoHiC is a available as docker container [39], or in the SaaS version on HPC4AI research cloud platform [40, 41] at http://neohic.hpc4ai.it. The latter version will become part of a more general portal , as [42]designed for sharing and analyse Hi-C data. Thanks to the Streamflow [43] HPC4AI native Workflow Management System, the portal will make it possible to define novel analysis “Pipeline-as-a-Service” and run them in the HPC4AI or other public cloud directly addressing the reproducibility challenge in genomic research.

The possibility to easily extend NeoHiC in a more general pipeline will make it possible to extend the current analysis with new ones, for example with a machine learning stage to estimate contacts that are not visible from Hi-C experiment but can be inferred from data. Specifically, we do believe that an artificial neural network can be used to formulate hypotheses about contacts while a variational autoencoder can be used to check contact consistency to perform link prediction and estimate hidden data.

An important area is that of rare genetic diseases: the visual inspection could provide clues of similarity or differences between phenotypes and treatment effects. Since NeoHiC can be coupled with the gene ontology and enrichment analysis bringing the possibility of deep phenotype stratification. Another area of future expansion is the physical measurement of the distances between genes that could come from FISH or microscopy.

We believe this software can operate for both biological research and for diagnostic analysis. In particular, our vision is an integrated system in which Electronic Health Record, a histological report from anatomic pathology, and multi-omics data should be integrated to provide doctors with an enriched description of the disease, therefore improving the diagnosis. From the multi-omics point of view, Hi-C data can supplement other information such as genome sequencing for the identification of particular genotypes, RNA-seq for profiling the transcriptomics activity of cells and epigenetic profiling, as shown in Fig. 9.

The visualisation capability provided by NeoHiC increases the real usability of Hi-C pipelines. Also, it could allow a further development as visual inference tool to study large scale chromatin in vitro effects of demethylating or nucleic acid intercalating drugs. The fusion of clinical, histology and omics data integration can be achieved using HPC pipelines, for data analysis, and machine learning approaches, to achieve a multi-modal data integration, providing reliable Clinical decision support system [44].

Our tool is among the first of a new fast-growing volume of applications in the field of graph databases for bioinformatics and medical informatics. This class will bring a revolution in bioinformatics as it makes methods and results more interpretable than existing methods.

## Supplementary Information

**Additional file 1.** This file describes how to generate the input data matrix for Neo4jstarting from Hi-C data from the GEO repository.

## Data Availability

NeoHiC is available online on the HPC4AI@UNITO.IT cloud at http://neohic.hpc4ai.it. The source code is available at https://github.com/dddagostino/neohic. The dataset used for the experiments is described in [34].

## Abbreviations

APOC: Awesome Procedures on Cypher
CSV: Comma Separated Values
ER: Estrogen Recept
ERGM: Exponential random graph models
FISH: Fluorescent in situ hybridization
Hi-C: High-throughput sequencing Chromosome Conformation Capture
HPC: High Performance Computing
MCMC: Markov chain Monte Carlo
SaaS: Software-as-a-Service
TAD: topologically associating domains
WNT: Wingless-related integration site
