# Supplementary material for: Advantages of using graph databases to explore chromatin conformation capture experiments
Source: BMC Bioinformatics. 2021 Apr 26;22(Suppl 2):43. doi: 10.1186/s12859-020-03937-0 (PMC8073886; doi:10.1186/s12859-020-03937-0)
Supplement: Supplementary file 1 — Additional file 1. This file describes how to generate the input data matrix for Neo4jstarting from Hi-C data from the GEO repository. [file 12859_2020_3937_MOESM1_ESM.pdf]

DATA GENERATION TUTORIAL

Data Acquisition and Preparation

We used as a test example of NeoHiC the breast cancer Hi-C dataset from Achinger-Kawecka et al. [1] that is publicly available at Gene Expression Omnibus, National Center for Biotechnological Information under the access number GSE130916. This dataset contains 6 Hi-C experiments, which were conducted in endocrine-sensitive breast cancer cells (MCF7) at three time-points during long-term culture: time zero (T0, two replicates), mid time point (T16, two replicates) and late time point (> 6 months, T32, two replicates).

In order to reproduce the results achieved in the paper, the first step of the pipeline consists in downloading data from GEO. This can be achieved using the sratoolkit software suite (https://www.ncbi.nlm.nih.gov/books/NBK158900/). In particular, the prefecth command can be used to directly download data related to a specific SRA project from the online repository and converting them to fastq.

```
./prefetch --type fastq SRR9030110
```

Identification of contacts

The fastq should be then aligned against the reference genome taking into account the peculiarity of the Hi-C experiments. In particolar, although libraries must be sequenced in paired-ends, the aligner must align reads as single-end in order to identify the different regions of the genome that are in close proximity. This can be achieved using hicup (https://www.bioinformatics.babraham.ac.uk/projects/hicup/), which relies on bowtie (http://bowtie-bio.sourceforge.net/index.shtml) as sequence aligner. Hicup performs a sequence of steps (hicup_truncating, hicup_mapping, hicup_filtering, hicup_deduplicating) that are essential to achieve high quality results. In particular, the software should discard all reads that are not compliant with the experimental protocol, according to the used restriction enzymes. To enforce this filter, a file with the in-silico digested genome, which can be achieved with the hicup_digester utility of hicup, must be provided as input to the software. All the parameters that define the Hi-C experiment and the desired filters threshold ashould be defined in a config file, which is provided as input to hicup.

```
./hicup -c config.txt
```

The output of hicup is a file in which valid couples of reads are reported with their mapping coordinates and quality. This file is usually provided as input to software that statistically analyze the contacts among the different regions of the genome, providing as output a contact matrix or a graph describing the 3D conformation of chromosomes.

NuChart

While many software provide as output a contact matrix, there are limited options for achieving a graph-based representation. In particular, we used NuChart to compute an undirected graph representation of the contact among genes in the genome. The software takes in input the the files generated by hicup with the valid read paris and some mandatory parameters describing how to compute the graph, such as the universe of genes to consider, the starting gene and the number of iterations of the algorithm. Other parameters can be specified to set the number of threads used in the computation, the output format and the desired plots.

```
./nuchart -C BRCA1 -S hicup.sam -N human_genes.txt -L 4 -P
```

By default, nuchart produces several output files, e.g. the degree distribution, the nodes degree and the list of edges.

NeoHIC

The files produced by NuChart have to be parsed for inserting them into the Neo4j graph databases and visualising with NeoHIC. In particular there is the need to process the file containing the list of edges, because it contains all the information required to add an experiment through NeoHiC. This is achieved by executing the *nuchart2neo4j.js* script.

Let us suppose we want to add data contained in the file *edges_Hi-C_MCF7Caldon_p32_a1_R1.csv*

We execute the script as

node nuchart2neo4j.js edges_Hi-C_MCF7Caldon_p32_a1_R1.csv T_32_Rep1

Where the last parameter, *T_32_Rep1* , represents the label that will be used to identify the experiment hereafter in NeoHIC.

The script produces three files: *T_32_Rep1.csv* containing statistics data on the experiment, *T_32_Rep1_edges.csv* containing the links found in this experiment and *T_32_Rep1_nodes*.csv containing the list of involved genes.

The files should be copied in the *import* directory of Neo4j and data insertion is performed using the Neo4j shell interface, *cypher-shell* contained in the *bin* directory, with the following commands

LOAD CSV WITH HEADERS FROM "file:///T_32_Rep1.csv" AS line
MERGE (n:experiment {label: line.name, nodes: toInteger(line.nodenum), relations: toInteger(line.edgenum), rootgene: line.rootgene});

LOAD CSV WITH HEADERS FROM "file:///T_32_Rep1_nodes.csv" AS line
MERGE(n:gene {label: line.name});

In the above commands the only parameter is represented by the name of the two files. It is to note that it is not necessary to insert the data contained in the node file if the full list of human genes has been previously inserted in Neo4j.

USING PERIODIC COMMIT 5000
LOAD CSV WITH HEADERS FROM "file:///T_32_Rep1_edges.csv" AS csvLine
MATCH (s { label: csvLine.start}),(q { label: csvLine.end})
CREATE (s)-[:T_32_Rep1 {weight: toFloat(csvLine.weight), wpath: toFloat(csvLine.wpath), prob: toFloat(csvLine.prob), link: toInteger(csvLine.link), ct: csvLine.ct }]->(q);

In this command, beside the name of the file, there is the need to specify the same label provided when executing *nuchart2neo4j.js*.

At this point the experiment labelled with *T_32_Rep1* will be shown in NeoHIC web interface for visualisation and analysis purpose.

EnrichR

Relying on the analysis that can be performed using NeoHiC different gene lists can be identified, such as, for example, genes that have cluster coefficient above a desired threshold or genes that belong to the same cluster community. These lists can be downloaded as CSV files from the NeoHiC interface and analysed using tools for enrichment analysis, a computational method for inferring knowledge about an input gene set by comparing it to annotated gene sets representing prior biological knowledge.

A very popular tool for enrichment analysis is EnrichR (https://maayanlab.cloud/Enrichr/). Enrichr takes as input a list of Entrez gene symbols and provides as output a data table with the name of the reference gene sets, p-values, z-scores, and combined scores. A bar plot is also provided as graphical representation, in which the length of the bar represents the significance of that specific gene-set or term and the color how much the term is significant.

[1] Achinger-Kawecka J, Valdes-Mora F, Luu PL, Giles KA et al. Epigenetic reprogramming at estrogen-receptor binding sites alters 3D chromatin landscape in endocrine-resistant breast cancer. Nat Commun 2020 Jan 16;11(1):320.
